# Segmentation of white blood cells and comparison of cell morphology by linear and naïve Bayes classifiers

**DOI:** 10.1186/s12938-015-0037-1

**Published:** 2015-06-30

**Authors:** Jaroonrut Prinyakupt, Charnchai Pluempitiwiriyawej

**Affiliations:** Electrical Engineering Department, Faculty of Engineering, Chulalongkorn University, Bangkok, Thailand

**Keywords:** White blood cell, Image segmentation, Ellipse curve fitting, Feature extraction, Classification

## Abstract

**Background:**

Blood smear microscopic images are routinely investigated by haematologists to diagnose most blood diseases. However, the task is quite tedious and time consuming. An automatic detection and classification of white blood cells within such images can accelerate the process tremendously. In this paper we propose a system to locate white blood cells within microscopic blood smear images, segment them into nucleus and cytoplasm regions, extract suitable features and finally, classify them into five types: basophil, eosinophil, neutrophil, lymphocyte and monocyte.

**Dataset:**

Two sets of blood smear images were used in this study’s experiments. Dataset 1, collected from Rangsit University, were normal peripheral blood slides under light microscope with 100× magnification; 555 images with 601 white blood cells were captured by a Nikon DS-Fi2 high-definition color camera and saved in JPG format of size 960 × 1,280 pixels at 15 pixels per 1 μm resolution. In dataset 2, 477 cropped white blood cell images were downloaded from CellaVision.com. They are in JPG format of size 360 × 363 pixels. The resolution is estimated to be 10 pixels per 1 μm.

**Methods:**

The proposed system comprises a pre-processing step, nucleus segmentation, cell segmentation, feature extraction, feature selection and classification. The main concept of the segmentation algorithm employed uses white blood cell’s morphological properties and the calibrated size of a real cell relative to image resolution. The segmentation process combined thresholding, morphological operation and ellipse curve fitting. Consequently, several features were extracted from the segmented nucleus and cytoplasm regions. Prominent features were then chosen by a greedy search algorithm called sequential forward selection. Finally, with a set of selected prominent features, both linear and naïve Bayes classifiers were applied for performance comparison. This system was tested on normal peripheral blood smear slide images from two datasets.

**Results:**

Two sets of comparison were performed: segmentation and classification. The automatically segmented results were compared to the ones obtained manually by a haematologist. It was found that the proposed method is consistent and coherent in both datasets, with dice similarity of 98.9 and 91.6% for average segmented nucleus and cell regions, respectively. Furthermore, the overall correction rate in the classification phase is about 98 and 94% for linear and naïve Bayes models, respectively.

**Conclusions:**

The proposed system, based on normal white blood cell morphology and its characteristics, was applied to two different datasets. The results of the calibrated segmentation process on both datasets are fast, robust, efficient and coherent. Meanwhile, the classification of normal white blood cells into five types shows high sensitivity in both linear and naïve Bayes models, with slightly better results in the linear classifier.

## Background

Blood smear images from a microscope provide important information for diagnosing and predicting diseases in haematological analysis. Blood samples are prepared and sent to a blood cell counter for calculating each type of cell. If haematologists find an unusual number of cells in any type, they will investigate further by looking into the microscopic blood smear, recount the number of cells and check their morphology in more detail. Any blood cells with irregular shapes or characteristics may trigger a presence of severe diseases. The visual inspection by haematologists is quite tedious and time consuming. Therefore, an automating process is highly desirable to accelerate the process. Three kinds of blood components present in a blood smear are red blood cells (RBCs), white blood cells (WBCs) and platelets. The RBCs transport oxygen from the lungs to all living tissues in the body and carry away carbon dioxide. They are normally found in up to 40–50% of the total blood volume. RBCs’ diameter is 6–8 μm. The WBCs play an important role in the body’s immune system by defending the body against both infectious disease and foreign materials. Therefore, analysis of WBC characteristics is essential.

Characterized by the presence of granules in their cytoplasm, WBCs can be classified into two groups (see Table 1). Basophil, eosinophil and neutrophil are granulocytes.

**Table 1:** Each type of WBC size, approx. % in adults and diameter [16]

Type	Granulocytes			Agranulocytes	
	Basophil	Eosinophil	Neutrophil	Lymphocyte	Monocyte
Microscopic image					
Approx. % in adults	0.4	2.3	62	30	5.3
Diameter (μm)	10–16	9–15	9–15	Small lymphocytes 7–8 Large lymphocytes 12–18	12–20

Basophil is responsible for allergic reaction and antigen. Basophil’s granules are of irregular distribution. Their large size appears dark-blue and visible on top of the nucleus that may obscure the cell nucleus. Eosinophil, playing a role in killing parasites, has lobed nuclei. Eosinophil’s granules are large, spherical and appear orange. Neutrophil is most abundant in the blood stream. It has multiple lobed nuclei. Neutrophil’s granules are defined by small red granules within blue cytoplasm resulting in lilac or pink colour. Lymphocyte and monocyte are agranulocytes. The texture of their nuclei is generally uniform. Meanwhile, the nucleus of the lymphocytes is round, that of the monocytes resembles a kidney. Nucleus and cytoplasm morphological features provide a means to identify and classify each type of WBCs. Other features, like size and approximated percentage found in adults, are also detailed in Table 1. The first part of this paper focuses on segmentations of the nucleus and the cytoplasm regions of the WBCs. The segmented results will be used to classify their types in the second part.

Much has been written on WBC segmentation. Ramoser et al. [1] used a set of features to describe cytoplasm and nucleus properties and applied pairwise support vector machine (SVM) classification to discriminate them. Fang et al. [2] implemented a fast WBC image segmentation using an on-line trained neural network. Their algorithm is based on the mean shift method and uniform sampling to reduce the training set while preserving the most distributed information. Bergen et al. [3] combined pixel-wise classification with template matching to locate erythrocytes and used the level-set approach to get exact leukocyte nucleus and plasma regions. Mohamed et al. [4] proposed an automatic blood cell nuclei segmentation based on grey scale contrast enhancement and filtering before removing false objects by finding the minimum size. Rezatofighi et al. [5] introduced another approach to WBC nuclear segmentation based on the orthogonality theory and Gram-Schmidt process. Sadeghian et al. [6] reviewed a framework for WBC segmentation. The framework is an integration of several digital image processing algorithms to segment nucleus and cytoplasm. Nucleus segmentation algorithms are based on morphological processing; whereas, cytoplasm algorithms are based on pixel intensity threshold. The limitation of this framework, however, is that only a small set of sub-images are tested. Ghosh et al. [7] implemented an automated approach to leukocyte recognition based on fuzzy divergence and modified thresholding techniques. They investigated the Gamma, Gaussian and Cauchy distributions of fuzzy membership functions through the segmented nuclei areas. It was found that Cauchy distribution provided the best segmentation results among the three. In addition, image thresholding was applied to improve the recognition rate.

There are also a few studies related to WBC classification. Yampri et al. [8] proposed the Eigen-face concept for the pre-classification of blood cell based on parametric feature detection. The derived Eigen-value and Eigen-vector provide the important features in the classification process. First, the WBC images are classified into two groups based on the number of nucleus lobes. Group A comprised eosinophil and neutrophil with only one nucleus. Group B included WBC with multiple nuclei. Group B was further divided according to the nuclei’s size. Monocyte (in group B1) had small nuclei; whereas, basophil and lymphocyte (group B2) had larger nuclei. Finally, the authors applied principal component analysis (PCA) to groups A and B2 to further classify each WBC within the groups. The training phase of the classifiers used a library of 50 WBC patterns. In the testing phase, they worked on 50 samples of data. The experiment was conducted on normal cells. The results showed a correct classification rate of about 92%. Rezatofighi et al. [9] introduced another approach to WBC classification. It is based on Gram–Schmidt orthogonalization, and they used the snake algorithm [10] to segment nucleus and cytoplasm. Then, they extracted various features from the segmented region and selected the most discriminative features using a Sequential Forward Selection (SFS) algorithm. Next, they compared the performances of two classifiers: Artificial Neural Network (ANN) and SVM. Extracted features were composed of the morphological features, e.g., nucleus and cytoplasm areas, nucleus and whole cell perimeters, the number of separated parts of the nucleus, means and variances of nucleus and cytoplasm boundaries, and the ratio between cytoplasm and nucleus areas. Texture features such as co-occurrence matrix and local binary patterns were also used. The co-occurrence matrix included 14 features representing contrast, homogeneity, entropy and other texture quantities.

Su et al. [11] proposed an idea to find the discriminating region of white blood cells in the Hue-Saturation-Intensity (HSI) colour space. The colours of each pixel in the discriminating region were considered as nucleus and cytoplasm of WBC. Then a morphological process was used to segment WBC. They extracted geometrical, colour, and LDP-based texture features from the segmented result. These features were used to classify five types of WBCs using three kinds of neural networks: multilayer perceptron, the SVM and the hyper rectangular composite neural networks. Tabrizi et al. [12] proposed to use Gram-Schmidth orthogonalization and the snake algorithm to segment nucleus and cytoplasm, respectively. They extracted three features from the segmented result. The best features were chosen through PCA. Finally, classification into five types of white blood cells was done with a Learning Vector Quantization (LVQ) neural network [13, 14]. 302 images in all were tested. Overall accuracy was about 96%. Theera-Umpon and Dhompongsa [15] showed that a nucleus alone could classify WBCs. They tested their algorithm with bone marrow images. The algorithm applies mathematic morphology to analyze WBC nucleus based features and uses naïve Bayes classifiers and ANNs with five-fold-cross validation. The result showed that features from the nucleus alone led to a 77% classification rate on average.

This paper presents a method to locate WBCs in microscopic blood smear samples and segment them into nucleus and cytoplasm regions. Features are extracted, and then SFS is applied to select a subset of features without any transformation. Finally, linear and naïve Bayes classifiers are employed to sort the cells into eosinophil, lymphocyte, monocyte and neutrophil. The performance of both classifiers is compared. The details of this proposed method are in “Methods”. Next section shows the experiment results and discussion. Final section is conclusion.

### Datasets

Blood smear microscopic images were collected from normal peripheral blood slides (dataset 1). The study’s algorithm was tested on 555 images (a total of 601 WBC) under a light microscope with 100× magnification captured by a high-definition color camera head Nikon DS-Fi2. All images were recorded and saved in JPG format of 960 × 1,280 pixels. The calibration ruler scale from the manufacturer was 10 μm equal to 150 pixels. In addition, a database of white blood cells downloaded from the CellaVision Competency Software (dataset 2) was tested for robustness. Dataset 2 had 477 images with a total of 477 WBCs. Each image was saved in JPG format of 360 × 363 pixels. The calibration scale was estimated from the size of RBCs to be 7 μm equal to 70 pixels.

For comparison, all images were also manually segmented into nucleus and cell (or cytoplasm) areas and classified into normal leukocytes: basophil, eosinophil, lymphocyte, monocyte and neutrophil by a hematologist.

## Methods

**Figure 1:**
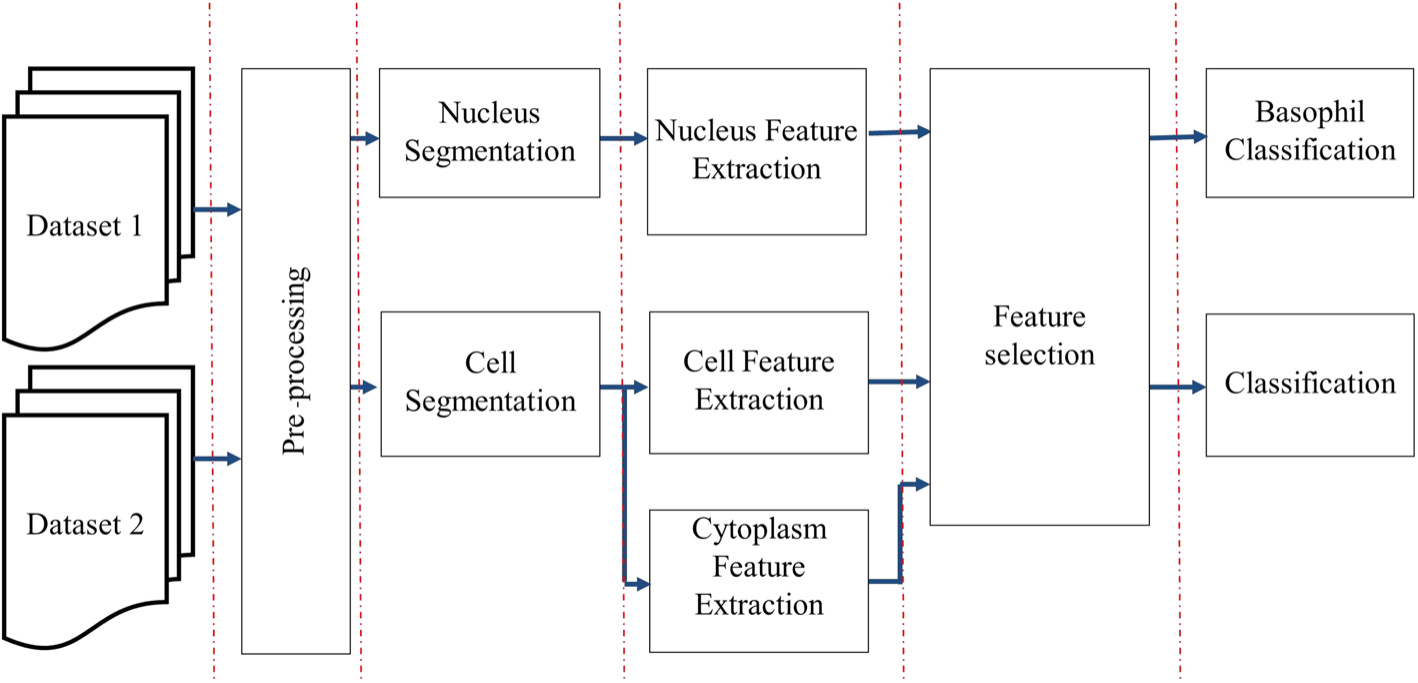
A block diagram of proposed system.

As shown in Figure 1, the proposed system could be divided into five main steps: pre-processing, segmentation, feature extraction, feature selection and classification.

### Pre-processing

**Figure 2:**
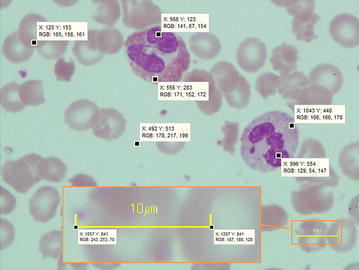
Pixel intensity among RGB channel in each area and zoom in calibration ruler.

**Figure 3:**
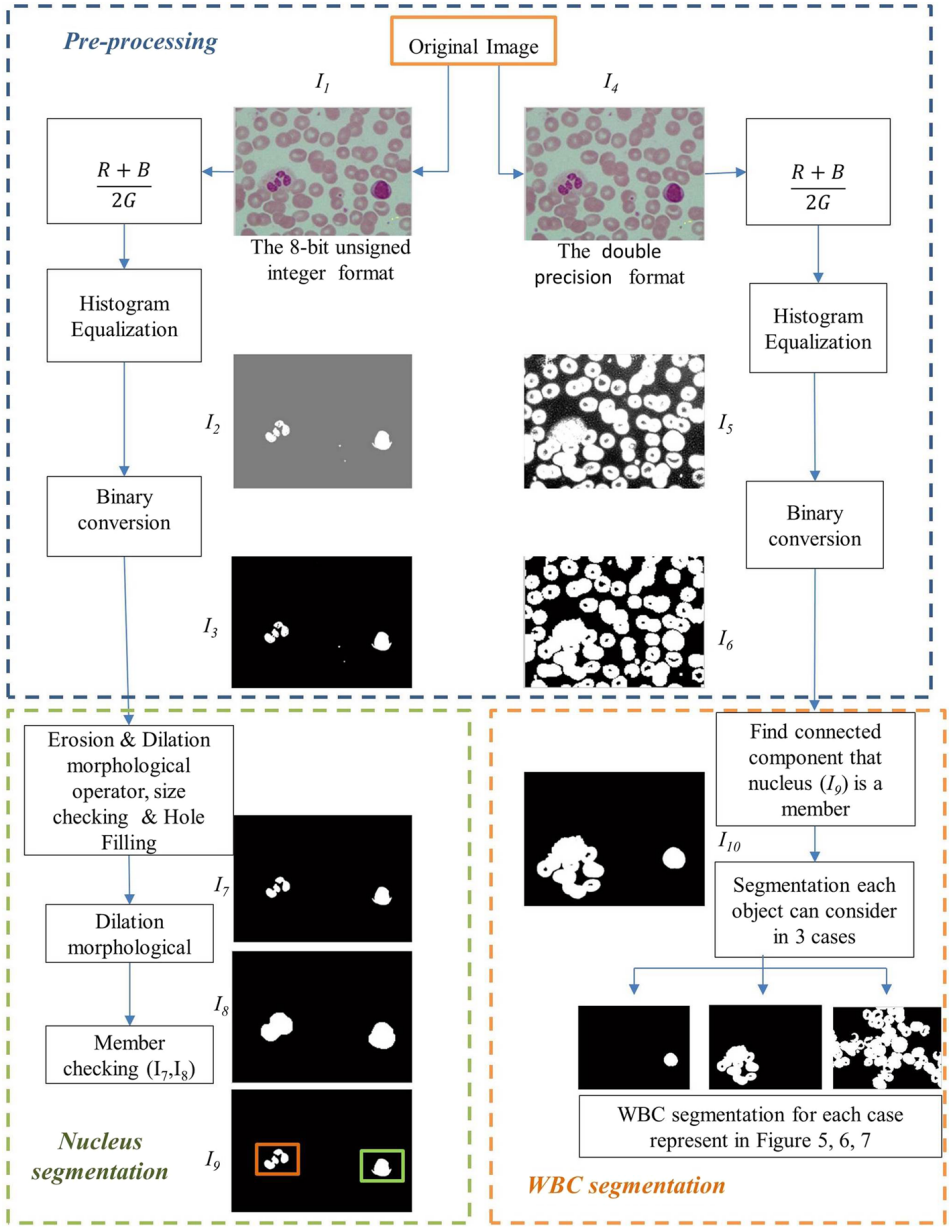
Block diagram of pre-processing step and segmentation process.

Since there are five different types of white blood cells based on their shapes, sizes and existence of granules, it is necessary and very challenging to segment the WBCs out of each image first. It is obvious that the nucleus of WBCs in all images appears in violet color. As shown in Figure 2, among the three RGB color components, the nucleus region in violet color has the least value in the green channel when compared to other regions such as cytoplasm or background. Therefore, the nucleus region was enhanced in the input images by averaging the pixel values in the red and blue channels together and then dividing the sum by the intensity value of the green channel. The process was conducted on images in both the 8-bit-unsigned integer and the double precision floating point formats, as shown in the block diagram of Figure 3. Histogram equalization was then applied to redistribute the image intensity to cover the whole intensity range of both images. Binary conversion was used to convert the image into binary format. The final image in the 8-bit-unsigned integer format image *I*_3_ was used as the nucleus enhanced image. The other, *I*_6_, was used as the WBC enhanced image.

### Nucleus segmentation

After the preprocessing step, erosion and dilation morphological operators with flat disk structuring element of radius 0.33 μm (dataset 1 is 5 pixels, dataset 2 is 3 pixels) was applied to the nucleus enhanced image to remove any noisy pixels.

**Figure 4:**
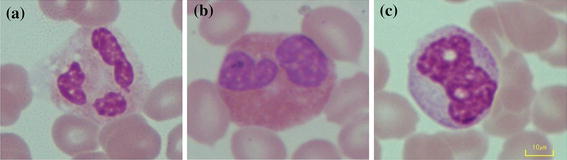
**a** Neutrophil with multilobes, **b** eosinophil with multilobes and **c** hole inside nucleus from phagocytosis process of WBCs.

Figure 4a, b approximate the minimum area of the nucleus by considering that only some types of WBCs have a nucleus with multiple lobes such as the eosinophil and the neutrophil. As shown in Table 1, although both of their diameters are in the same range of 9–15 μm, eosinophil nucleus usually has two lobes separated by a very narrow filament or stand, while the neutrophil nucleus has two to five lobes separated by very narrow filaments or stands. According to [16], the nucleus area to cytoplasm area ratio of the neutrophil is approximately 1:3. The nucleus area is a quarter of the total minimum area of the WBC. In this experiment, it was assumed that the estimated nucleus of neutrophil would be separated equally into 3 lobes. The minimum area for each lobe can be calculated as follows:

Total minimum area of neutrophil = (9 × 10^−6^ m)^2^ = 8.1 × 10^−11^ m^2^

Nucleus area $$= Total\, cell\, area / 4\; = 20.25 \times 10^{ - 12} \,{\text{m}}^{2}$$

Minimum nucleus lobe area $= Nucleus \,area/3 \; = \; 6.75 \times 10^{ - 12}\, {\text{m}}^{2}$

Minimum nucleus lobe area $= \;6.75 \times 10^{ - 12}\, {\text{m}}^{2} = (2.6 \times 10^{ - 6}\, {\text{m}})^{2}$

*Calculation of minimum nucleus lobe area in pixel unit from image resolution*

For dataset 1, image resolution is 150 pixels/(10 × 10^−6^ m)

Minimum nucleus lobe area $= \left( {2.6 \times 10^{ - 6} \,{\text{m}} \times \frac{{ 150\, {\text{pixels}} }}{{ \left( {10 \times 10^{ - 6}\, {\text{m}}} \right)}}} \right) ^{2} = (39\, {\text{pixels}})^{2}$

Minimum nucleus lobe area $= (39 \,{\text{pixels}} \times 39\, {\text{pixels}}) = 1,521 \,{\text{pixels}}$

For dataset 2, image resolution is $70\, {\text{pixels}} /\left( {7 \times 10^{ - 6}\, {\text{m}}} \right)$

Minimum nucleus lobe area $= \left( {2.6 \times 10^{ - 6}\, {\text{m}} \times \frac{{70\, {\text{pixels}} }}{{ (7 \times 10^{ - 6} {\text{m}})}}} \right)^{2} = (26\, {\text{pixels}})^{2}$

Minimum nucleus lobe area $= 26\, {\text{pixels}} \times 26\, {\text{pixels}} = 676\, {\text{pixels}}$

A minimum nucleus lobe area at 1,500 pixels and 670 pixels for dataset 1 and 2, was used respectively. Sometimes, a hole did exist inside the nucleus, (see Figure 4a), caused by the phagocytosis process of the WBCs; therefore, any holes with an area under 1.49 μm × 1.49 μm (500 pixels in dataset 1 and 220 pixels in dataset 2) was filled. The segmented multi-lobe nucleus could appear as multiple cells next to each other, which could lead to misinterpretation. Therefore, morphology dilation was applied with a flat disk structuring element of radius 2 μm (28 pixels for images in dataset 1 and 20 pixels for images in dataset 2) to merge them into one cell.

### White blood cell segmentation

The problems found in WBC segmentation are its variety of shapes and sizes. Moreover, the color of the WBC’s cytoplasm is indistinguishable from adjacent RBCs, making it even more challenging. Further processing is needed for more accurate results. Since cytoplasm surrounds the entire nucleus, it was assumed that all pixels connected to the nucleus pixels are candidates for the WBC. They can possibly be either white blood cell alone or white blood cell adjacent to other cells.

The method to separate adjacent cells is done under the assumption that the nucleus is in the center of the WBC. Therefore, the radial lines were created from the center of the convexed nucleus to the boundary of the object of interest as described by Eqs. (1) and (2)

1 $$\left[ {\begin{array}{*{20}c} {y_{1} } \\ {y_{2} } \\ \end{array} } \right] = \left[ {\begin{array}{*{20}c} 1 & {x_{1} } \\ 1 & {x_{2} } \\ \end{array} } \right]\left[ {\begin{array}{*{20}c} {a_{0} } \\ {a_{1} } \\ \end{array} } \right],$$

2 $$y\left( x \right) = \frac{{x - x_{2} }}{{x_{1} - x_{2} }}y_{1} + \frac{{x - x_{1} }}{{x_{2} - x_{1} }}y_{2 },$$

where $\left( {x_{1} ,y_{1} } \right)$ is the coordinate of the center of the convexed nucleus and $\left( {x_{2} ,y_{2 } } \right)$ is the coordinate at each position on the boundary of the object of interest.

**Figure 5:**
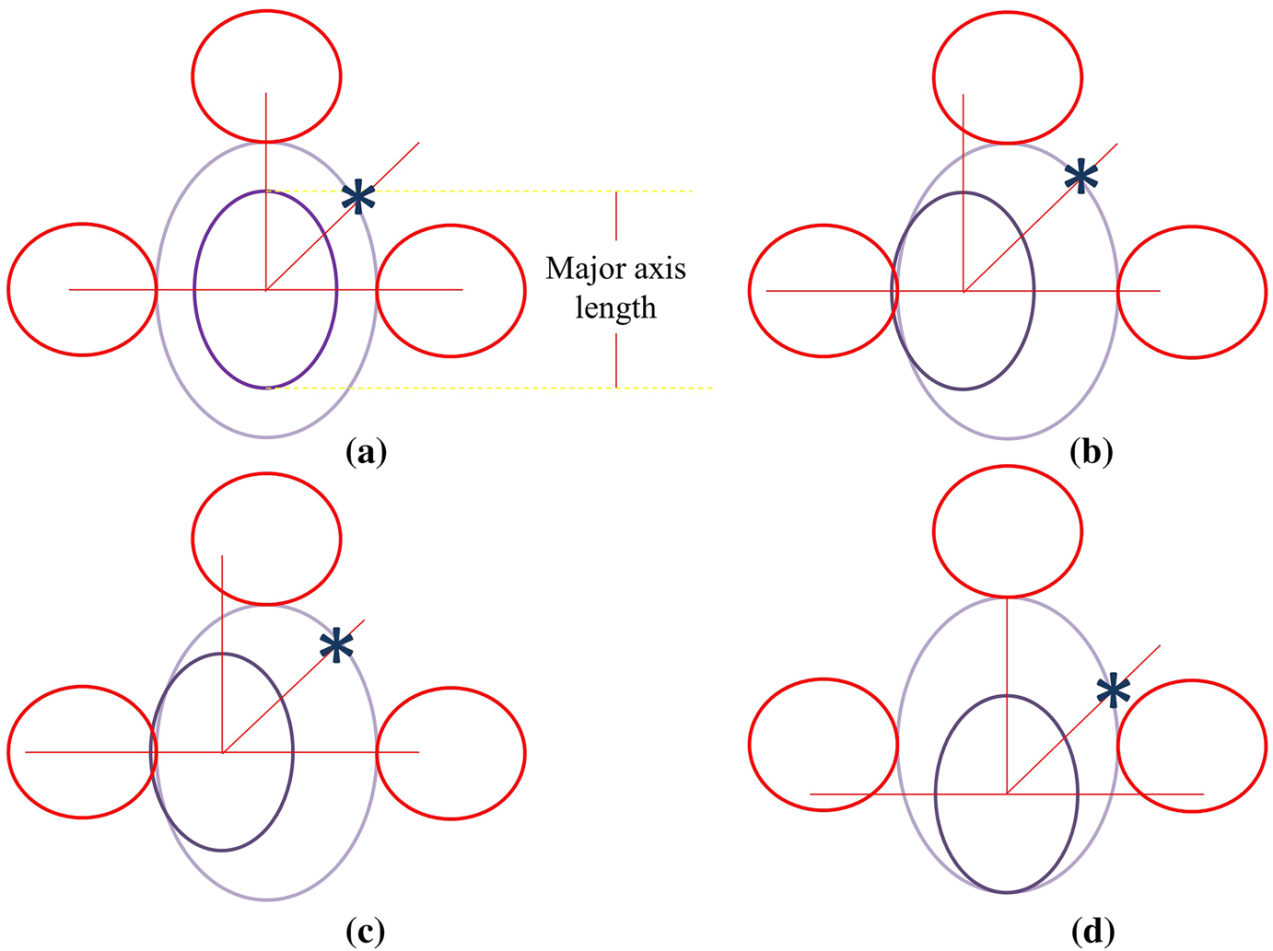
The estimate model when the *lilac line* represents the nucleus boundary, the *blue line* represents the WBC boundary, the *red line* represents the RBC boundary and the radial line is the line which originates from the centre of the nucleus and length equals the major axis length of convex nucleus. **a**–**d** Represent the different position of the nucleus while the *blue star* is the candidate point of cell boundary.

The total number of white pixels along each line representing the length of each radial line should be approximately of the same value. Sometimes, a nucleus is not at the center of the cell and the selected object may not be a single cell, see Figure 5b–d. To handle these problems, a convex hull of the selected nucleus object was created to avoid the case of multi-lobe nucleus. Then, the convex hull nucleus image was subtracted from the selected object. Next, radial lines were drawn on the subtracted image at the centroid of the convex hull to the boundary of the selected object. Finally, the total number of white pixels along each radial line were counted and a threshold value was set as the average value of the total white pixels found from each line with additional 2  μm (30 pixels for dataset 1 and 20 pixels for dataset 2) to compensate for the case when the nuclei are not at the center. If the number of peaks was more than the threshold value, it was considered a part of adjacent cells.

There were then three possible outcomes: (1) the whole object is identified as WBC if no adjacent cells exist, (2) the object is next to some other cells, in which case, further segmentation is required and (3) the object’s area is more than 20% of the whole image area (estimated from the ratio between maximum possible area in case 2 to the total area). In this case, morphological dilation was applied with a disk structuring element of radius 6 μm, or the minimum diameter of RBCs within the segmented nucleus. Finally, the ‘AND’ operation was used to combine the object of interest with the dilated image. Only part of the object, when segmented nucleus is a member, was considered in the next segmentation step.

Since RBCs are the most prevalent component found in blood smear images, its diameter can be estimated from a calibration ruler. Each WBC has a nucleus, cytoplasm and some granules. Edge detector alone can hardly distinguish the real WBC boundary. However, the outer edge of interested object can be assumed to be the boundary of white blood cell if its radial lines from the center of the nucleus are less than the major axis of the convex nucleus. This study’s models when the nucleus is not at the center of the WBC are illustrated in Figure 5. Each radial line follows the equations

3 $$\begin{aligned} x_{2} &= x_{1} + r\cos \theta ,\; \hfill \\ y_{2} &= y_{1} + r\sin \theta , \hfill \\ \end{aligned}$$

where point $\left( {x_{1} ,y_{1} } \right)$ is the center of the convex nucleus image, point $\left( {x_{2} ,y_{2} } \right)$ is the end point of the radial line, $r$ is the length of the major axe of the convex nucleus image, and $\theta$ is the angle of radial line for $\theta = \left[ {0, 0.1, 0.2, \ldots, 2\pi } \right]$.

**Figure 6:**
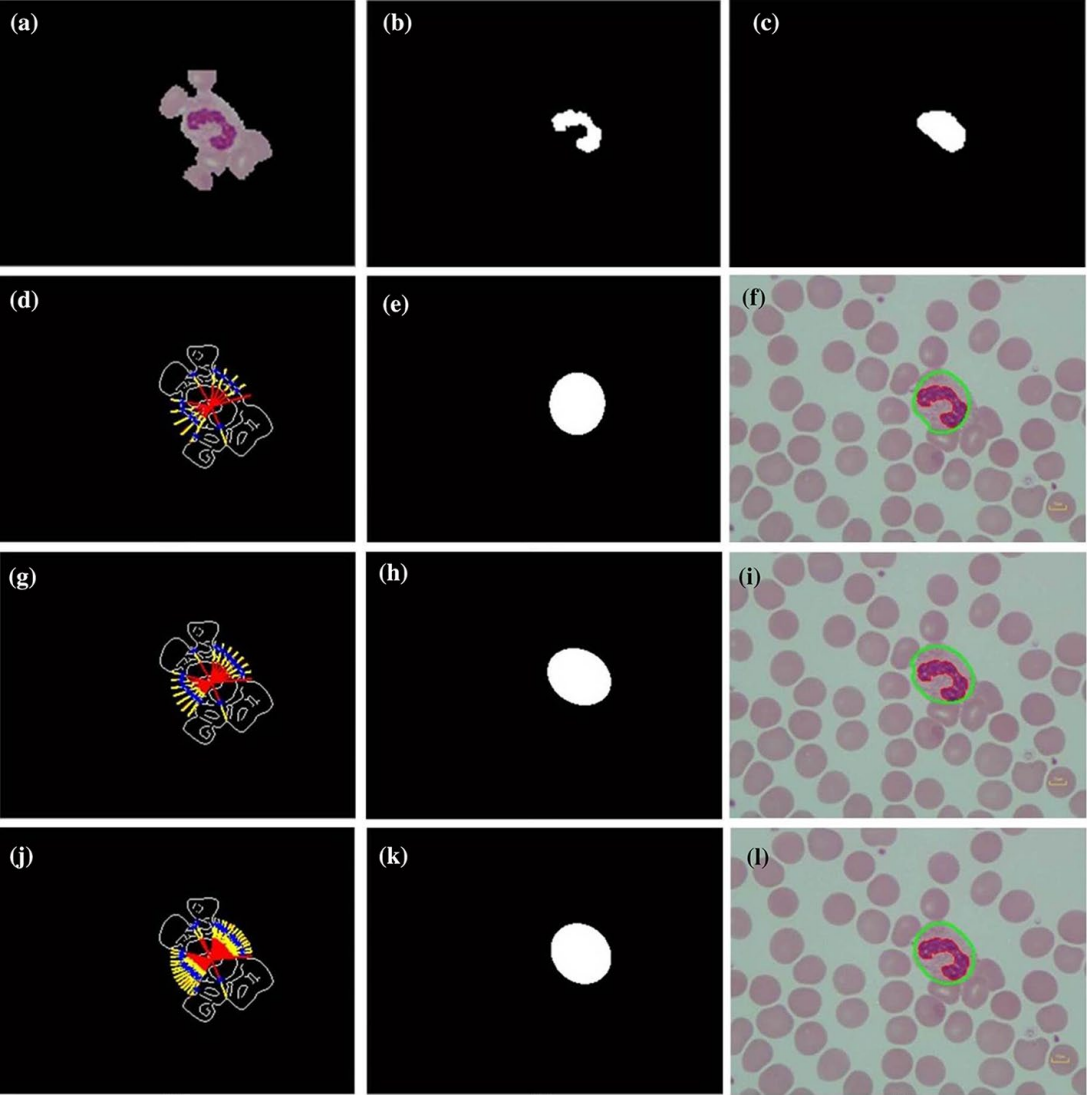
Cell segmentation step. **a** Selected object in RGB, **b** segmented nucleus, **c** convex hull of nucleus region, **d** radii line with centre at the centroid of convex hull, radius equal to major axis length, angle between radii line is 0.3 rad, *blue point* is the candidate point, **e** applied direct least square for ellipse fitting to the candidate point, **f** segmented result: the *red contour* is the nucleus region, the *green contour* is the cell region, **g**–**i** and **j**–**l** are the repeated step of **d**–**f** with angle between radii line of 0.2 and 0.1 rad respectively.

First, the Canny edge algorithm was applied to create an edge candidate image. Then, radial lines with radius equal to the length of the major axis of the convex object was drawn with the origin on the boundary of the convex hull image. The points under consideration had to follow two conditions: They were at the end of the intersection between the edge point and the radial line, and the end point of the radial line in the selected object in RGB image, see Figure 6a, must have zero intensity value. Any edge points on the radial lines, shown as blue points in Figure 6d, g, j, were candidate cell edges. More radial lines provided finer segmented results. The angles between radius lines were 0.3, 0.2 and 0.1 rad, respectively. Next, the direct least square fitting for an ellipse shape proposed by Fitzgibbon et al. [17] was applied to these candidate points. With the obtained ellipse parameters, the boundary of the WBC could be lineated. The ‘AND’ operation was then applied to the estimated area of nucleus and final cell segmentation. The results are as shown in Figure 6k. Finally, cytoplasm region was also segmented by subtracting the segmented cell area from the segmented nucleus area.

### Feature extraction and selection

The criteria used to extract meaningful features are granule existence, the number of nucleus lobes, color intensity and variance values in nucleus and cytoplasm, the difference in smear color values and the size of cell or nucleus. A large number of features could be extracted from both the nucleus and cell segmentation results. Some interesting features used in this paper are mean intensity, variance, number of concave points, area, area ratio, perimeter, roundness, entropy and intensity ratio. However, only features that have high correlation with the class prediction were selected. In this paper, SFS technique was applied to choose suitable features.

#### Features extracted from binary segmented image

Nucleus and cell segmented regions are a connected area in binary image $O_{i} , i > 0$, as in Eq. (4)

4 $$O_{i} \left( {x,y} \right) = \left\{ {\begin{array}{*{20}c} {1, \quad for\, points\, on\, the\, object} \\ {0,\quad for\, background\, points} \\ \end{array} } \right..$$

The area $A_{i}$ of the *i*th object ($O_{i}$) is the total number of pixels as in Eq. (5)

5 $$A_{i} = \mathop \sum \limits_{x = 0}^{M - 1} \mathop \sum \limits_{y = 0}^{N - 1} O_{i} \left( {x,y} \right).$$

The area ratio is computed from the ratio of the nucleus area to the cell area.

The perimeter $P_{i}$ of the object $O_{i}$ can be calculated by extracting the edge image of the object and counting the total number of pixels on the edge image.

The roundness of the object $O_{i}$ can then be calculated from Eq. (6)

6 $$R_{i} = \frac{{4 \pi A_{i} }}{{P_{i}^{2} }}.$$

#### Features extracted from statistical data based on histogram

The mean intensity of the gray values in each *R*, *G* or *B* channel of the segmented image follows Eq. (7)

7 $$m = \mathop \sum \limits_{j = 0}^{L - 1} r_{j} p\left( {r_{j} } \right),$$

$r_{j}$ is the *j*th gray level, which has a probability as $p\left( {r_{j} } \right)$.

To calculate the variance,

8 $$\sigma^{2} = \mathop \sum \limits_{j = 0}^{KL - 1} \left( {r_{j} - m} \right)^{2} p\left( {r_{j} } \right).$$

Entropy describes the complexity within the image; an image with complex scene has high entropy. The equation is as in Eq. (9)

9 $${\text{entropy}} = - \mathop \sum \limits_{j = 0}^{L - 1} p\left( {r_{j} } \right) {\text{log}}_{2} \left[ {p\left( {r_{j} } \right)} \right].$$

**Figure 7:**
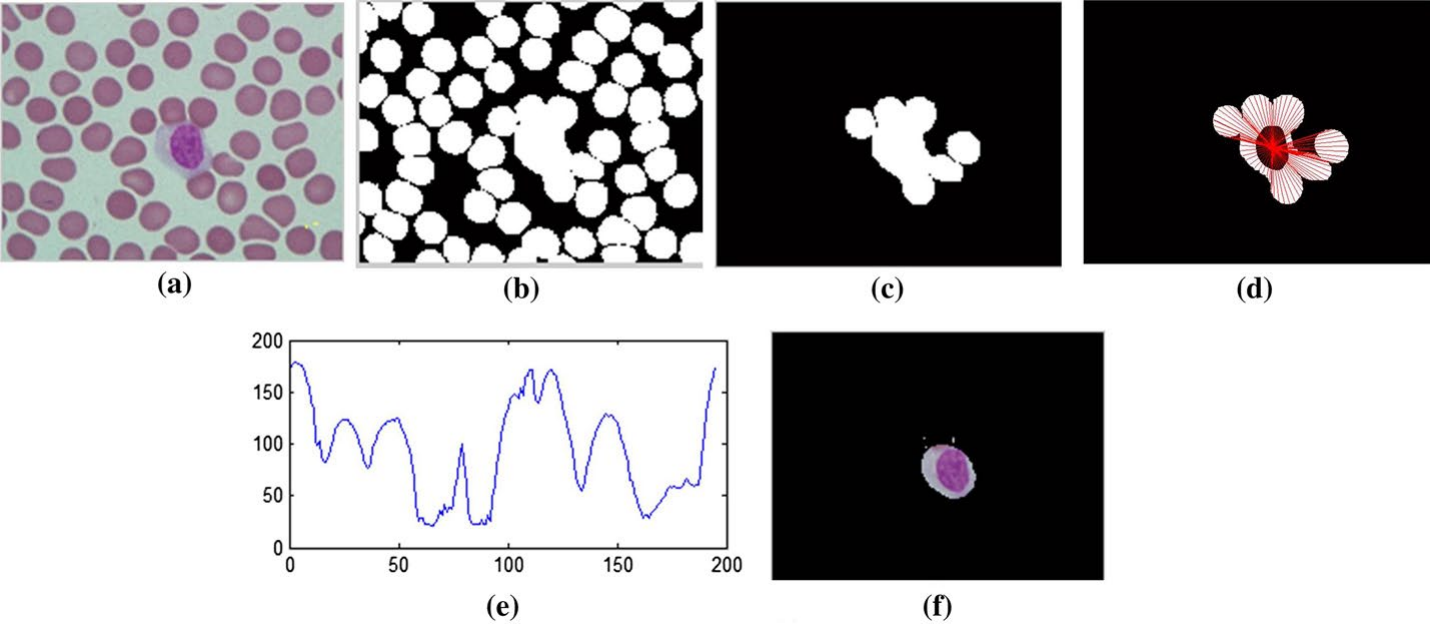
The interested object in the second case. **a** Original image, **b** preprocessing image, **c** interested object, **d**, **e** concave checking and **f** segmented cell.

The number of concavities is found by subtracting the segmented nucleus image from its convex hull. As in Figure 7d, the total number of intersecting points and the threshold level divided by two is the total number of concavities.

The mean intensity ratio is the ratio between two colors in each region. This study considers the mean intensity ratio the *G* channels of the segmented nucleus and cell areas. The mean intensity ratio between the *R* and *B* channels of segmented cytoplasm area segmented area was also used.

This study employed 15 features as follows:

1. mean intensity values of the red channels in the nucleus area,
2. variance of the intensity values of the red channels in the nucleus area,
3. mean intensity values of the green channels in the nucleus area,
4. variance of the intensity values of the green channels in the nucleus area,
5. mean intensity in cytoplasm area in green channel,
6. variance of intensity values of the green channels in cytoplasm area,
7. the number of concave points found in nucleus area,
8. the ratio between nucleus and cell areas,
9. nucleus area,
10. cell roundness,
11. entropy of nucleus in blue channel,
12. entropy of cell in red channel,
13. the ratio of mean intensity value in the red and blue channel in cytoplasm area,
14. the ratio of mean intensity value in red and green channel in cell area,
15. the ratio of mean intensity value in green and red channels in the nucleus area.

### Classification

The result of the segmented nucleus area can be used to identify the basophil. As stated earlier that basophil has granules all over the whole cell and its area is more than 100 × 10^−12^ m^2^, this study estimated the minimum diameter of the basophil as shown in Table 1, (22,500 pixels and 10,000 pixels in area for dataset 1 and 2, respectively) which is larger than all other granulocytes but between the monocyte and large lymphocyte; whereas, the intensity values in the red and blue channels have variance higher than both agranulocyte. So, nucleus area, the variance of intensity in red and blue channels of the segmented area, classify to basophil. Cell segmentation to basophil can be a convex hull of the nucleus.

Forward feature selection was used to choose significant features. However some features had a different range compared with others. Therefore, they needed to be normalized before any classification. This was done by unit vector normalization.

Next, linear classifier was used to recognize each type of WBC and then compare the classification results with naïve Bayes classifier. Since the total number of images for each cell type could vary, the tenfold Leave One Out technique was used for cross validation testing.

## Results

### Segmentation results

**Figure 8:**
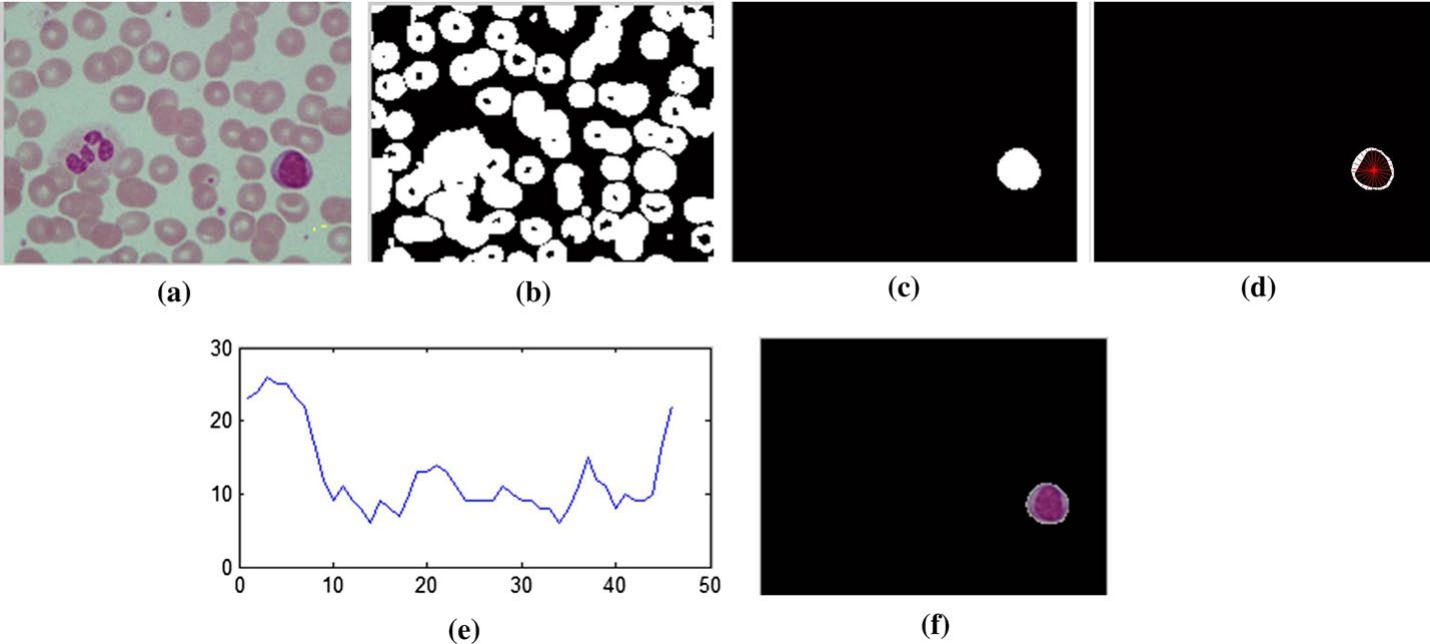
The interested object in the first case. **a** Original image, **b** preprocessing image, **c** interested object, **d**, **e** concave checking and **f** segmented cell.

**Figure 9:**
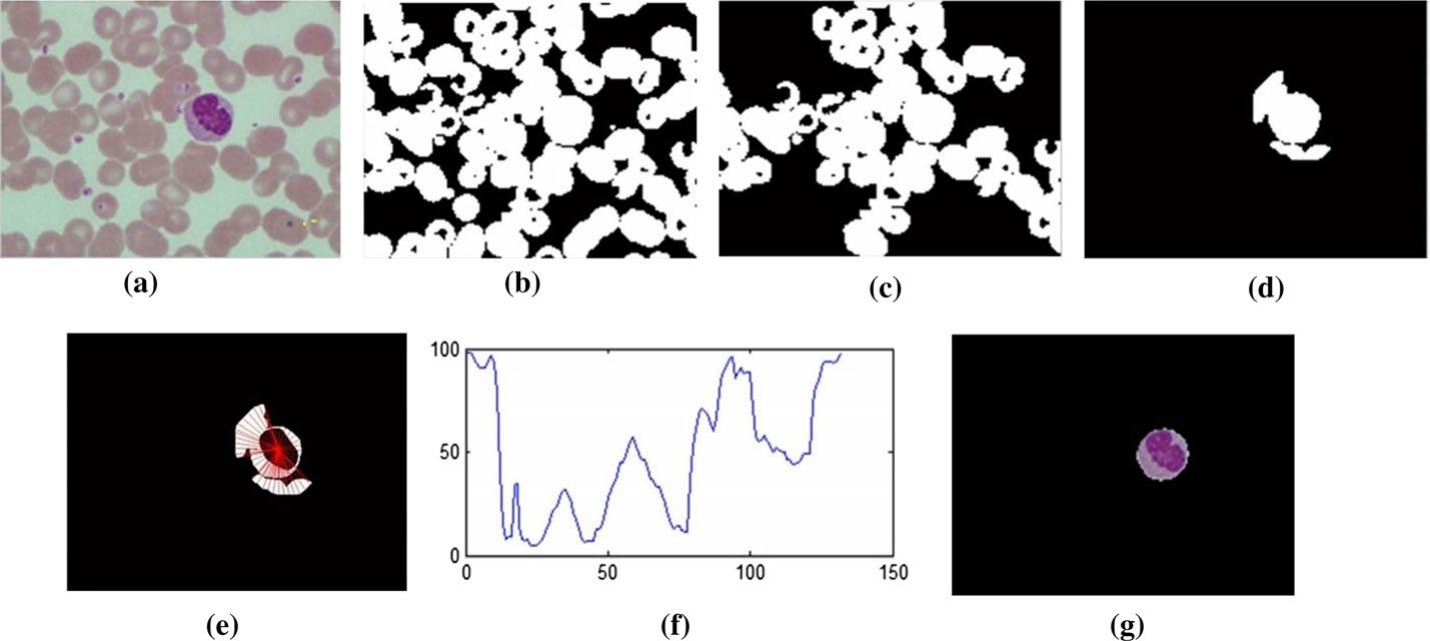
The interested object in the third case. **a** Original image, **b** preprocessing image, **c** interested object, **d** dilate nucleus area, **e**, **f** concave checking and **g** segmented cell.

The 8-bit-unsigned integer and the double precision format images are shown in *I*_1_ and *I*_4_ of Figure 3. The image after histogram equalization, as shown as *I*_2_ of Figure 3, depicts a quite distinct nucleus area while image *I*_5_ of Figure 3 covers all RBC, and the cytoplasm area of WBCs and the dark region represents the background. The images *I*_7_, *I*_8_ and *I*_9_ in Figure 3 represent the results from nucleus segmentation. The image *I*_10_ is the selected object composing WBC. The selected object is one of the three cases as shown in *I*_11_, *I*_12_ and *I*_13_. The interested object in three cases is shown in Figures 7, 8, and 9 respectively. Figure 6d, g, j show candidate cell edges. More radial lines would provide finer segmented results. In this figure, angles between radial lines are 0.3, 0.2, and 0.1 rad. A 0.1 rad angle was used between radial lines for segmenting.

The segmentation results are shown in Figure 6. The nucleus region is lineated by the red line. The whole WBC boundary is drawn in green, and the candidate edge points are masked by the blue stars.

**Figure 10:**
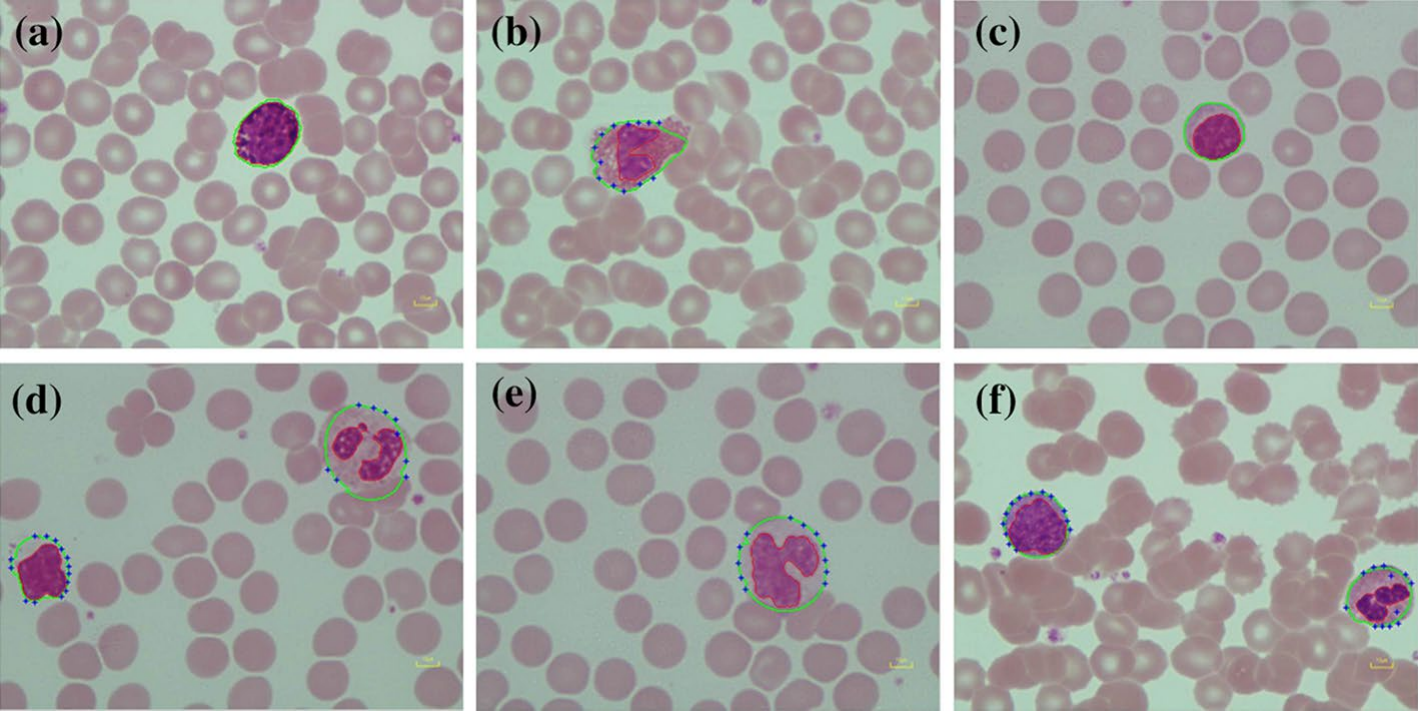
Segmentation result of various WBC from dataset 1. **a** Basophil, **b** eosinophil, **c** lymphocyte, **d** lymphocyte and neutrophil, **e** monocyte and **f** lymphocyte and neutrophil.

**Figure 11:**
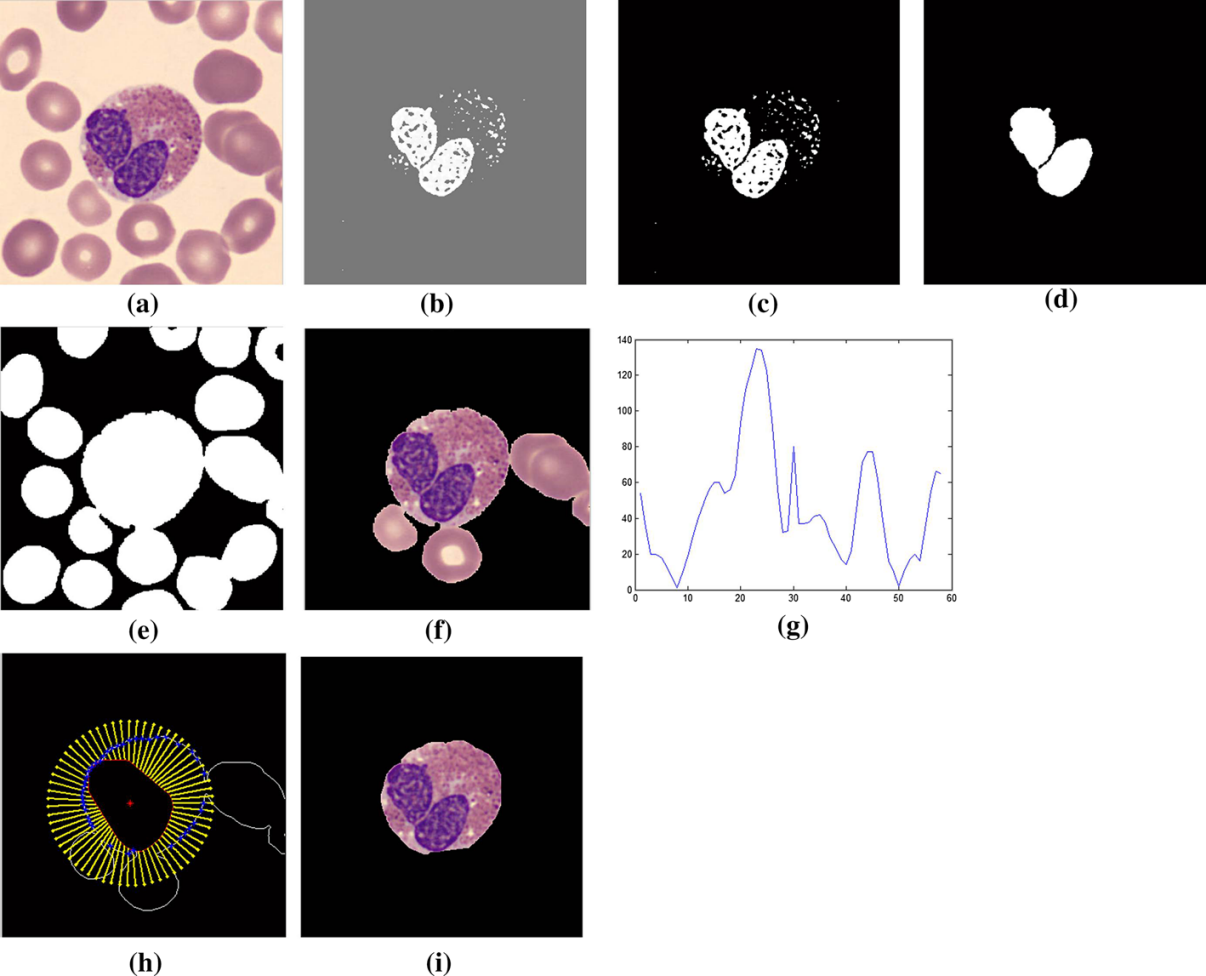
All algorithm processes from dataset 2. **a** Original image, **b**, **c** nucleus segmentation, **d** segmented nucleus area, **e**, **f** cell segmentation with interested area, **g** concave consideration, **h** candidate point finding and **i** segmented cell area.

This segmentation method was tested on 601 WBCs in 555 digital microscopic images collected from the Pathology Unit, Biomedical Science Department, Faculty of Science, Rangsit University and WBC images downloaded from the CellaVision Competency database. The number of cells characterized by each WBC type in both datasets are shown in Table 2. The study’s algorithm was test on both datasets with some parameters set according to different image resolutions and image sizes. The segmentation results of various WBCs from dataset 1 are shown in Figure 10. The overall step of this algorithm from dataset 2 is presented in Figure 11.

**Table 2:** Dataset

Type	Basophil	Eosinophil	Lymphocyte	Monocyte	Neutrophil	Total
The digital microscopic image (dataset 1)	5	9	175	38	374	601
CellaVision dataset (dataset 2)	1	5	158	42	271	477
Total	6	14	333	80	645	1,078

This algorithm can find the WBC position correctly. The performance of the segmentation results was then evaluated by comparing the segmented area from the proposed algorithm with the manual segmentation performed by the haematologist. Similarity measures based on regional overlapping dice similarity, false positive ratio (FPR) and false negative ratio (FNR) were used. The definitions and metrics to report the results of this study are as follows:

10 $${\text{Dice similarity}} = \frac{{2{\text{TP}}}}{{2{\text{TP}} + {\text{FP}} + {\text{FN}}}},$$

11 $${\text{False positive ratio}} = \frac{\text{FP}}{{{\text{TN }} + {\text{FP}}}},$$

12 $${\text{False negative ratio}} = \frac{\text{FN}}{{{\text{TP}} + {\text{FN}}}},$$

where true positive (TP) is the number of cell pixels of interest correctly identified as cell pixels of interest, false positive (FP) is the number of non-interesting cell pixels that are incorrectly identified as cell pixels of interest, true negative (TN) refers to the number of non-interested cell pixels that are correctly identified as non-interesting pixels and false negative (FN) is the number of cell pixels of interest that are incorrectly identified as non-interesting cell pixels.

**Figure 12:**
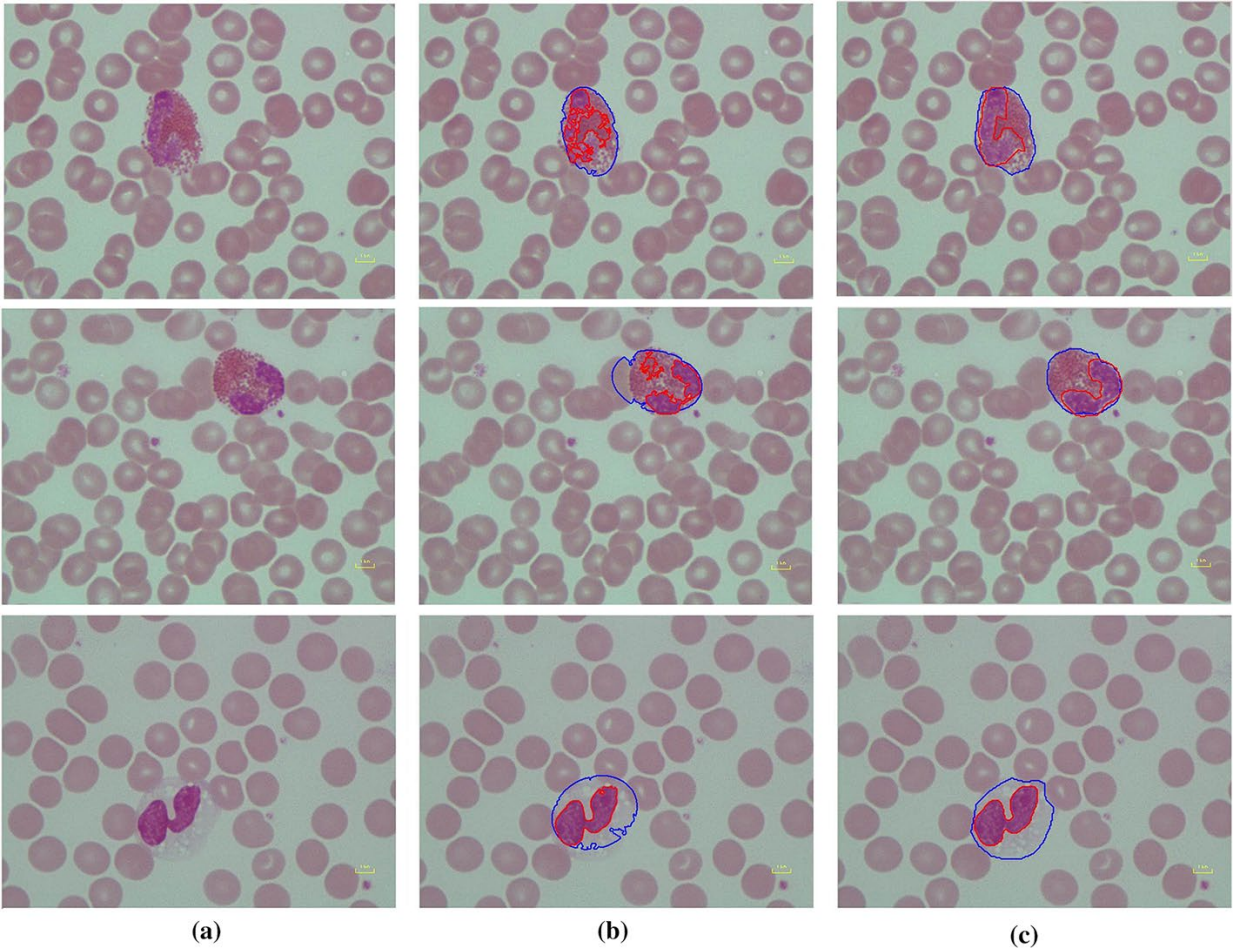
Error of segmentation on dataset 1. When row(1) and row(2) are eosinophil, row(3) is monocyte, column **a** original image, **b** segmented result, **c** haematologist segmented result: *red line* is nucleus boundary and *blue line* is cell boundary.

**Figure 13:**
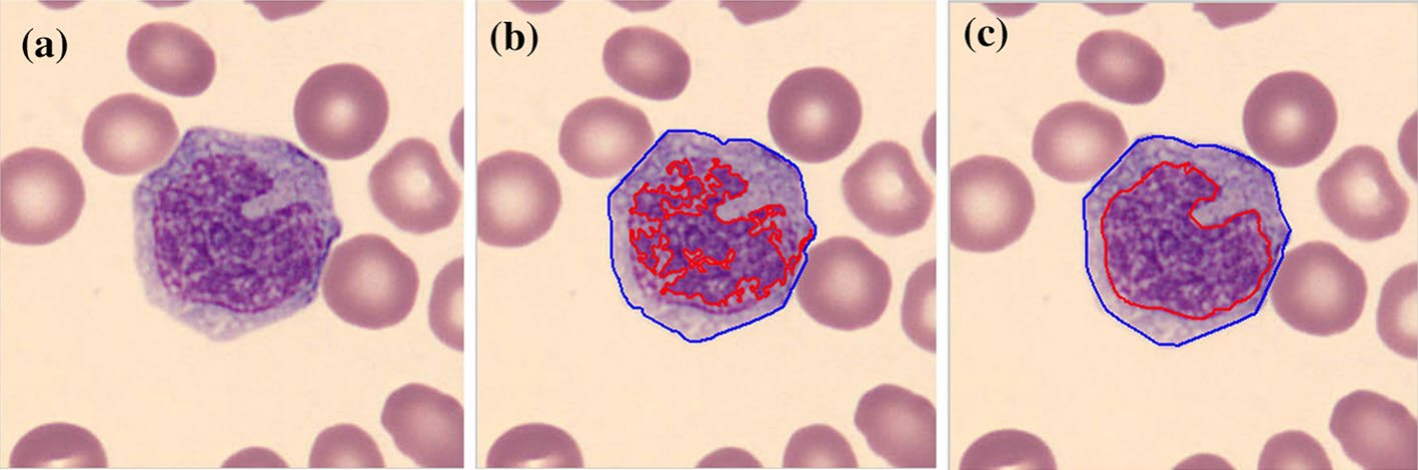
Error of segmentation on monocyte for dataset 2. **a** Original image, **b** Segmented result, **c** haematologist segmented result: *red line* is nucleus boundary and *blue line* is cell boundary.

Table 3, shows that the dice similarity for all types of WBC in both nucleus and cell segmentations has values more than 82%. This verifies that the proposed segmentation algorithm provides very good results in agreement with the manually segmented gold standard. However, it was found that some eosinophils in dataset 1 had granules that cover the whole cell, see Figure 12 row (1). This may lead to some nucleus area segmentation error. Moreover, the lowest value of 0.825 dice similarity for monocyte cell segmentation in dataset 1 could be caused by its transparent cytoplasm which is indistinguishable from background, see Figure 12 row (2). Otherwise, it seem that the lowest value of 0.875 nucleus similarity for monocyte nucleus segmentation in dataset 2 may cause a fine chromatin pattern and cytoplasmic vacuoles as shown in Figures 12 and 13, while cell segmentations in dataset 2 have values over than 0.913.

**Table 3:** Performance of segmentation

Type	The digital microscope collected image (dataset 1)						CellaVision dataset (dataset 2)					
	Baso	Eos	Lym	Mono	Neu	Average	Baso	Eos	Lym	Mono	Neu	Average
Nucleus segmentation												
Dice similarity	0.950	0.868	0.977	0.940	0.948	0.937	0.881	0.923	0.972	0.875	0.995	0.929
FPR	0.002	0.163	0.002	0.000	0.002	0.034	0.205	0.131	0.056	0.025	0	0.083
FNR	0.094	0.098	0.042	0.108	0.094	0.087	0.051	0.037	0.005	0.193	0.009	0.059
Cell segmentation												
Dice similarity	0.955	0.836	0.919	0.825	0.899	0.887	0.913	0.947	0.963	0.945	0.966	0.947
FPR	0.008	0.275	0.072	0.225	0.089	0.134	0.002	0.092	0.052	0.061	0.038	0.049
FNR	0.078	0.100	0.089	0.146	0.112	0.105	0.159	0.023	0.023	0.052	0.030	0.057

The average of FPR on nucleus and cell segmentation of dataset 1 is 0.034 and 0.134, respectively. The average of FNR on nucleus and cell segmentation of dataset 1 is 0.087 and 0.105, respectively. The average of FPR and FNR of dataset 2 was lower than dataset 1 for both nucleus and cell segmentation since most of image has the WBC with no connected cell.

### Classification results

The confusion matrix of basophil classification, which is classified in nucleus segmentation part, is shown in Table 4.

**Table 4:** Confusion matrix of basophil classification

	Basophil (predict)	Non-basophil
Basophil (actual)	6	0
Non-basophil (actual)	1	1,071

Other WBCs are classified into four types: eosinophil, lymphocyte, monocyte and neutrophil, with selected features described above. This study’s model was tested with the Leave-One-Out approach, one type of cross validation technique, with tenfolds. The average correction rate of linear classifier is 0.976 (the average error rate equal to 0.034); whereas, the average correction rate of naïve Bayes classifier is 0.941 (the average error rate is 0.059). The average confusion matrix of this study features on a linear classifier and naïve Bayes classifier as shown in Tables 5 and 6, respectively.

**Table 5:** Confusion matrix of our experiment feature on linear classifier

	Eosinophil (predict)	Lymphocyte (predict)	Monocyte (predict)	Neutrophil (predict)
Eosinophil (actual)	1	0	0	0
Lymphocyte (actual)	0	32	1	0
Monocyte (actual)	0	0	8	0
Neutrophil (actual)	0	0	1	63

**Table 6:** Confusion matrix of our experiment feature on naïve Bayes classifier

	Eosinophil (predict)	Lymphocyte (predict)	Monocyte (predict)	Neutrophil (predict)
Eosinophil (actual)	1	0	0	0
Lymphocyte (actual)	0	31	0	2
Monocyte (actual)	0	1	7	0
Neutrophil (actual)	1	1	1	62

The model assigned to the correct class was evaluated to the test samples by calculating the accuracy, sensitivity, specification and precision from the confusion matrix using the following equation:

13 $${\text{Accuracy }} = \frac{{({\text{TP }} + {\text{TN}})}}{{({\text{TP}} + {\text{TN}} + {\text{FP}} + {\text{FN}})}},$$

14 $${\text{Sensitivity }} = \frac{\text{TP}}{{({\text{TP }} + {\text{FN}})}},$$

15 $${\text{Specificity }} = \frac{\text{TN}}{{ ({\text{TN}} + {\text{FP}})}},$$

16 $${\text{Precision }} = \frac{\text{TP}}{{({\text{TP}} + {\text{FP}})}},$$

where TN is the number of correct predictions of negative instance, FP is the number of incorrect predictions of positive instance, FN is the number of incorrect predictions of negative instance, and TP is the number of correct predictions of positive instance.

The accuracy, sensitivity, specification and precision of basophil classifications are 99.8, 100, 99.8 and 85.7%, respectively. The average values of accuracy, sensitivity, specificity and precision of the linear model are 98.7, 98.1, 99.5 and 89.2%, respectively. The average value of accuracy, sensitivity, specificity and precision of naïve Bayes model are 97.3, 96, 98.8 and 80.6%, respectively. The details are shown in Tables 7 and 8.

**Table 7:** Accuracy, specification and precision of eosinophil, lymphocyte, monocyte and neutrophil from linear classifier

Linear classifier	Eosinophil	Lymphocyte	Monocyte	Neutrophil	Average
Accuracy	0.997	0.984	0.977	0.988	0.987
Sensitivity	1.000	0.976	0.962	0.984	0.981
Specificity	0.997	0.996	0.985	1.000	0.995
Precision	0.750	0.991	0.833	0.995	0.892

**Table 8:** Accuracy, specification and precision of eosinophil, lymphocyte, monocyte and neutrophil from naïve Bayes classifier

Naïve Bayes classifier	Eosinophil	Lymphocyte	Monocyte	Neutrophil	Average
Accuracy	0.991	0.962	0.974	0.963	0.973
Sensitivity	1.000	0.940	0.935	0.966	0.960
Specificity	0.991	0.975	0.986	1.000	0.988
Precision	0.444	0.969	0.837	0.973	0.806

## Discussion

The study’s proposed system was tested on two datasets. In the segmentation process, some parameters needed to be adjusted depending on image resolutions and sizes. However, the segmentation results on both datasets are similar, implying that the study’s algorithm is robust. For the classification process, the extracted features of both datasets were merged together and then, the model was tested with the linear and naïve Bayes classifiers. The study used tenfold-leave-one-out cross validation, as the correction rate on average is highly satisfactory for both linear and naïve Bayes classifiers. This shows that the proposed model may overcome an over-fitting problem.

## Conclusions

The proposed WBC segmentation method that has been applied to two datasets, and the results are compared to the gold standard segmented manually by a haematologist. Both provide over 90% accuracy. This method is fast, robust and efficient. Consequently, white blood cell morphological characteristics can be extracted and used in linear and naïve Bayes classifiers for performance comparison. The linear classifier shows slightly better performance than the naïve Bayes one. In addition, the five types of white blood cells can be classified with high sensitivity.

It should be noted that the size of the images in dataset 2, downloaded from CellaVison software, is not from a standard camera. It is obvious that they have been cropped to cover only the white blood cell. However, resolution has been estimated based on real RBC size. Nonetheless, the testing on two image datasets with different resolutions shows that the proposed segmentation process can be calibrated to carry out different image sizes or formats as long as the resolution is known.
